# A protocol for a systematic review of non-randomised evaluations of strategies to improve participant recruitment to randomised controlled trials

**DOI:** 10.1186/s13643-016-0308-3

**Published:** 2016-08-02

**Authors:** Heidi R. Gardner, Cynthia Fraser, Graeme MacLennan, Shaun Treweek

**Affiliations:** Health Services Research Unit, University of Aberdeen, Foresterhill Health Campus, Health Sciences Building, Aberdeen, Scotland UK

**Keywords:** Clinical trial, Participant recruitment, Recruitment interventions, Patient selection, Recruitment strategies

## Abstract

**Background:**

Randomised controlled trials guard against selection bias and therefore offer the fairest way of evaluating healthcare interventions such as medicinal products, devices and services. Recruitment to trials can be extremely difficult, and poor recruitment can lead to extensions to both time and budget and may result in an underpowered study which does not satisfactorily answer the original research question. In the worst cases, a trial may be abandoned, causing huge waste. The evidence to support the choice of recruitment interventions is currently weak. Non-randomised evaluations of recruitment interventions are currently rejected on grounds of poor methodological quality, but systematic evaluation and assessment of this substantial body of work (using Grading of Recommendations Assessment, Development and Evaluation (GRADE) where possible) may provide useful information to support and inform the recruitment decisions of trialists and the research priorities of methodology researchers.

**Methods:**

The following databases will be searched for relevant studies: Cochrane Methodology Register, MEDLINE, EMBASE, CINAHL and PsycINFO. Any non-randomised study that includes a comparison of two or more interventions to improve recruitment to randomised controlled trials will be included. We will not apply any restrictions on publication date, language or journal. The primary outcome will be the number of individuals or centres recruited into a randomised controlled trial. The secondary outcome will be cost per recruit. Two reviewers will independently screen abstracts for eligible studies, and then, full texts of potentially relevant records will be reviewed. Disagreements will be resolved through discussion. The methodological quality of studies will be assessed using the Cochrane risk of bias tool for non-randomised studies, and the GRADE system will be used if studies are pooled.

**Discussion:**

This review aims to summarise the evidence on methods used to improve recruitment to randomised controlled trials. Carrying out a systematic review including only data from non-randomised studies is a novel approach, and one which some may argue is futile. However, we believe that the systematic evaluation of what is likely to be a substantial amount of research activity is necessary, worthwhile, and will yield valuable results for the clinical trials community regardless of whether the outcomes find in favour of one or more interventions.

Should the results of this review suggest that non-randomised evaluations do have something to offer trialists planning their recruitment strategies, the review may be combined in the future with the Cochrane review of randomised evaluations to produce a full review of recruitment strategies encompassing both randomised and non-randomised evaluation methods.

**Systematic review registration:**

PROSPERO CRD42016037718

**Electronic supplementary material:**

The online version of this article (doi:10.1186/s13643-016-0308-3) contains supplementary material, which is available to authorized users.

## Background

There is a peculiar paradox that exists in trial execution – we perform clinical trials to generate evidence to improve patient outcomes; however, we conduct clinical trials like anecdotal medicine: (1) we do what we think works; (2) we rely on experience and judgement and (3) limited data to support best practices [1].

Randomised controlled trials (RCTs) are at the core of evidence-based healthcare. They guard against selection bias and therefore offer the fairest way of evaluating healthcare interventions [2], whether these involve medicinal products, devices or services. Recruitment of both clinicians and participants to RCTs can be extremely difficult.

If recruitment fails to successfully reach target sample size of the trial, the trial may be deemed ‘underpowered’. Underpowered trials raise an important ethical question; participants have voluntarily taken part, they have been exposed to an intervention with unknown or uncertain benefit based on the premise that they are doing so for good reason, and yet, there is now the possibility that the research question will remain unanswered even after the trial has been completed.

The issue of poor recruitment is widespread; anecdotal evidence tells us it is often the most frustrating stage in the trial process, while more rigorous evidence suggests that <50 % of trials meet their recruitment target or meet their target without an extension [3–7]. More recent research has also highlighted the problem of frequent extensions to recruitment time. An examination of recruitment into trials taking place between 2002 and 2008 and funded by two of the largest funding bodies in the UK, the National Institute of Health Research (NIHR)’s Health Technology Assessment (HTA) programme and the UK Medical Research Council (MRC) [8], found that 45 % of trials received an extension of some kind, and these were no more likely to reach recruitment targets than those not receiving extensions.

This systematic review builds on the current Cochrane systematic review of interventions to improve trial recruitment [9] but with one substantial difference. Until now, systematic review of non-randomised studies of recruitment interventions has been largely rejected on the grounds that the evidence these studies provide with regard to intervention effect is of low quality. Carrying out a systematic review including only data from non-randomised studies is a novel approach, and one which some may argue is futile because of the low methodological quality of these studies for measuring effect. However, we believe that the systematic evaluation of what is likely to be a substantial amount of research activity is necessary (because without collation, it is currently ignored), worthwhile (because there may be substantial/promising undiscovered effects), and will yield valuable results for the clinical trials community regardless of whether the outcomes find in favour of one or more interventions. Moreover, we will use the Grading of Recommendations Assessment, Development and Evaluation (GRADE) [10] system of rating confidence in cumulative estimates, which means that non-randomised studies (typically viewed as low or very low quality) could have their quality rating increased if the aggregated data show large effects, a dose response or if all plausible confounders would have reduced the effect. This provides the potential for evidence from non-randomised studies to supplement RCT evidence, which is currently limited and often of low quality: only 12 of 45 included studies in the Cochrane recruitment review are considered to be at low risk of bias.

It is possible that our systematic and considered evaluation of this substantial body of research activity finds that it has little or no utility, in which case we will be in a strong position to recommend to funders and others that researchers stop designing, conducting and reporting such studies and spend their scarce resources doing something more beneficial.

What will we know after doing the review that we do not know now?

The Cochrane review of randomised evaluations of recruitment interventions has rejected dozens of non-randomised studies of recruitment interventions, meaning there is currently an untapped body of evidence that may help trialists make more evidence-based decisions about recruitment. Our review has the potential to deliver one of two results:

1. Collated non-randomised data provide help to trialists through two mechanisms:
  - At least some non-randomised studies can be pooled and their results are such that the GRADE rating for this body of evidence can be increased.
  - Trends in effect may suggest interventions worthy of more rigorous randomised evaluations.
2. Alternatively, the collated data from non-randomised studies remains of ‘low’ or ‘very low’ quality, displays no clear trends and is therefore of no use to trialists planning their recruitment strategies.

Both results would move the field forward.

Should we find result 1, we will open up a previously overlooked evidence base that trialists can use to inform their trial recruitment decisions. The nature of interventions tested using non-randomised studies is unclear, so there is a real possibility that we may reveal completely new, effective interventions which can considered by trialists. In the case of known recruitment strategies, we may uncover data that adds weight to existing interventions previously investigated through the use of RCTs. This would strengthen our current knowledge base. Some SMS-based interventions, for example, may fall into this category.

There is also the possibility that grouping of non-randomised studies may reveal encouraging trends, but methodological weaknesses do not allow for the GRADE rating to be raised. In this case, the work would suggest that these particular interventions are worthy of evaluation in an RCT, addressing current gaps in evidence. The subsequent use of well-designed RCTs would provide high-quality evidence to support or refute the suggested trend from the non-randomised studies. Moreover, the protocols for such RCTs could be published as Studies Within A Trial (SWATs) [11], meaning that trialists and others (including funders) have easy access to a database of recruitment interventions in need of evaluation. Finally, if we find result 1, we will discuss approaches to combining this review with the current Cochrane review of randomised evaluations of interventions to improve recruitment to trials (which ST leads) [9] with the Cochrane Methodology Review Group.

Should we find result 2, meaning that the data demonstrate consistently low quality with no evident trends, we will be able to say that there is very little point in continuing to invest our time, energy or money as a research community, in doing (and publishing) these non-randomised studies, which would then represent a recurring example of research waste.

Why should non-randomised evaluations of recruitment strategies be reviewed separately to the existing Cochrane review of randomised evaluations of recruitment strategies?

This review of non-randomised evaluations may provide little evidence of immediate use to trialists, despite a large number of included studies. As discussed previously, one possible use of this review is to make clear that non-randomised evaluations of recruitment interventions are of little use. Given this, and the length of time a combined review would take, it is prudent to deal with the non-randomised evidence separately to begin with. The two reviews may be combined at a later date, and the overlap in author teams means that this review can be completed with an eye on this.

## Objectives

The objective of this study is to quantify the effects of strategies to improve recruitment of participants to RCTs evaluated using non-randomised designs.

## Methods

### Types of studies

Non-randomised studies include a comparison of two or more interventions to improve recruitment to randomised controlled trials. We define ‘non-randomised studies’ as any quantitative study estimating the effectiveness of a recruitment intervention that did not use randomisation to allocate participants to intervention or comparison groups. These types of studies are referred to by multiple names in the literature including but not limited to observational studies, cohort studies and case-control studies.

Studies covering questionnaire response and studies of retention strategies will be excluded on the grounds that these are separate issues addressed by their corresponding Cochrane Methodology reviews [12, 13].

### Participants

Individuals involved in a trial. The context of the trial is likely to be healthcare but may not be, for the reason that interventions that are effective in other fields may also be applicable to settings in the healthcare environment. Strategies evaluated within simulated trials (studies that ask potential participants whether they would take part in a trial if it was to be run but the study does not run the trial) will not be eligible.

### Types of intervention

Any intervention or approach aimed at improving or supporting recruitment of participants nested within studies performed for purposes unrelated to recruitment. Included interventions could be wide-ranging, aimed at research ethics committees (e.g. interventions supporting the case for non-requirement of mandatory signed and witnessed consent for recruitment to a trial), collaborators (e.g. healthcare professionals recruiting patients for a trial) or study participants (e.g. patients being randomised to a trial). Examples of such interventions are the use of SMS appointment reminders for participants supplying a mobile telephone number, use of newspaper or social media advertising, a dedicated email address or a toll-free telephone number as the initial point of contact from interested participants, simplified consent procedures for participants within a particular age range and recruitment coordinators working at selected trial sites.

### Types of comparator

Any approach aimed at improving or supporting recruitment of participants nested within studies performed for purposes unrelated to recruitment. Examples are as given in the ‘Types of intervention’ section. The comparator could also be nothing, meaning the intervention is compared against taking no special measures to improve recruitment.

### Types of outcome measures

Primary: Number of individuals or centres recruited into a randomised controlled trial.

Secondary: Cost of using the recruitment intervention per recruit.

### Search methods for identification of studies

The search strategy was developed by an Information Specialist (CF), who has expertise in developing search strategies for health technology assessment, especially through being the Information Specialist attached to the Aberdeen-based National Institute of Health Research Technology Assessment Review Group (http://www.nets.nihr.ac.uk/programmes/hta/policy-makers/tar-teams). The strategy was reviewed by the other authors. To identify studies, we will search bibliographic databases and hand-search reference lists of both relevant systematic reviews and all included studies. We will search systematic reviews of randomised recruitment interventions (e.g. [9]) for studies that were excluded on the grounds that they were not randomised.

We will apply neither language nor time restriction.

We will search the following databases:

Cochrane Methodology Register (CMR)

Medical Literature Analysis and Retrieval System Online (MEDLINE), OVID 1946 to present

MEDLINE In-Process, OVID current

Excerpta Medica dataBASE (EMBASE), OVID 1947 to present

Cumulative Index to Nursing and Allied Health Literature (CINAHL), EBSCO 1981 to present

PsycINFO, Ovid 1967 to present

The following multifile search strategy for MEDLINE and EMBASE (OVID) will be adapted for the other databases listed.

- 1 *Patient selection/
- 2 *informed consent/
- 3 ((participat$ or recruit$ or enter$) adj4 (trial? or research or study or studies or random$)).tw.
- 4 (participat$ adj4 (recruit$ or decide$ or decision or agree$ or consent$)).tw.
- 5 1 or 2 or 3 or 4
- 6 exp *clinical trials as topic/use prmz
- 7 exp *“clinical trial (topic)”/use emcz
- 8 *research subjects/use prmz
- 9 *research subject/use emcz
- 10 recruit$.ti.
- 11 informed consent.ti.
- 12 (recruit$ adj7 (trial? or research or study or studies)).ti.
- 13 (participa$ adj7 (trial? or research or study or studies)).ti.
- 14 or/6–13
- 15 5 and 14
- 16 controlled clinical trial.pt. use prmz
- 17 controlled clinical trial/use emcz
- 18 intervention studies/
- 19 randomized controlled trials as topic/use prmz
- 20 “randomized controlled trial (topic)”/
- 21 experiment$.tw.
- 22 quasi-experimental study/ use emcz
- 23 impact.tw.
- 24 intervention?.tw.
- 25 chang$.tw.
- 26 evaluation studies/
- 27 evaluat$.tw.
- 28 effect$.tw.
- 29 examin$.tw
- 30 compar$.tw.
- 31 comparative studies/
- 32 or/16–31
- 33 exp animals/ not humans/
- 34 32 not 33
- 35 15 and 34
- 36 35 not (randomized controlled trial.pt. or randomi?ed.ti.
- 37 36 not randomized controlled trial/use emcz
- 38 37 not (letter or editorial or comment or abstract).pt.

### Data management

All search results will be merged into the reference management software EndNote, and duplicate records of the same report will be removed using the EndNote de-duplication tool. We will use a master spreadsheet to track all inclusions/exclusions, which will allow us to create a Preferred Reporting Items for Systematic Reviews and Meta-Analyses (PRISMA) flow diagram once the screening process is complete (Additional file 1). Extracted data will be collected on specially designed forms (see ‘Data extraction’ section) and data entered into Cochrane RevMan tool (http://tech.cochrane.org/revman) when the data make this possible.

### Identifying studies

Two authors will independently screen the titles and abstracts of all records retrieved from searches of the electronic bibliographic databases stated above. The full text will be acquired for studies that look as if they meet the inclusion criteria. The full texts of all potentially eligible studies will be independently reviewed by two authors to determine if they meet the stated inclusion criteria. We will seek additional information from study authors where necessary to resolve questions about eligibility and provide other data as required. Any disagreements throughout the process of trial identification and selection will be resolved through discussion and, if necessary, the involvement of a third reviewer.

### Risk of bias of individual studies

As we are using non-randomised studies only, the likelihood of increased heterogeneity resulting from residual confounding is heightened. The Risk Of Bias In Non-randomized Studies - of Interventions (ROBINS-I; https://sites.google.com/site/riskofbiastool/) will be used to assess the risk of bias due to confounding. Aspects of methodological quality such as participant selection, measurement of intervention, departures from intended interventions, missing data, measurement in outcomes and selection of the reported result in included non-randomised studies will be assessed using the ROBINS-I tool [14] (see https://sites.google.com/site/riskofbiastool/). Each study will be rated as critical, serious, moderate or low risk of bias based on a judgement of the gathered information. If there is insufficient detail reported in the study, the risk of bias will be classified as ‘no information’ and the original study authors will be contacted for more information. Reporting of information on the flow of participants through the trial (e.g. from a Consolidated Standards of Reporting Trials (CONSORT) diagram) will be recorded.

Data on risk of bias will be presented in an additional table for all included studies, and results will be interpreted in light of risk of bias; studies will not be excluded on the grounds of risk of bias. Where it is appropriate to do a meta-analysis, risk of bias will be included in the GRADE assessment of study limitations.

### Data extraction

A data extraction form will be developed to collect the outcome measures given under the ‘Types of outcome measures’ section. Data will be extracted from each article by two authors independently. Disparities in data extraction will be resolved through discussion and, if necessary, the involvement of a third reviewer. We will contact the authors of reports of potentially relevant studies to try and obtain information or additional data needed for the review that is not available in the published reports. Data will be extracted on the recruitment intervention evaluated, country in which the study was conducted, type of population, details on the study setting, description of study to be recruited into and numbers and proportions recruited using each intervention and comparator.

### Data synthesis

Trials will be analysed according to the type of intervention used in the study (e.g. newspaper articles, use of SMS reminders); interventions will be grouped when their form or content is deemed sufficiently alike. A further categorisation by type of participant will be used if we find the same intervention applied to more than one type of participant (e.g. patients, staff at recruiting centres).

### Dealing with missing data

Attempts will be made to contact study authors to obtain any missing data (e.g. participant, intervention or outcome details). Analyses will be conducted on an intention-to-treat basis where possible; alternatively, data will be analysed as reported. Loss to follow-up will be reported and assessed as a potential source of bias in our risk of bias assessment.

### Assessment of heterogeneity

The likely nature of the included studies means that we anticipate that much of the analysis will be narrative description of the data rather than a statistical analysis, which we intend to present in tables, grouped by intervention type (and possibly participant if the same intervention is used with different types of participant). Where population, intervention and outcome are sufficiently similar to allow pooling of data in a meta-analysis, we will look for both visual evidence of heterogeneity in forest plots and statistical evidence of heterogeneity using the chi-square test for heterogeneity and the degree of heterogeneity quantified using the *I*^2^ statistic [14]. Where substantial heterogeneity is detected (*I*^2^ ≥ 50 %), possible explanations will be investigated informally and the data summarised using a random-effects model if appropriate.

### Assessment of reporting bias

We will investigate reporting (publication) bias for the primary outcome using a funnel plot where 10 or more studies of the same population, intervention and outcome are available. Care will be taken when interpreting any asymmetry in the funnel plots as this is not always due to publication bias.

### Confidence in cumulative estimate

Where possible, we will pool studies and apply the GRADE approach to give an overall assessment of the certainty of the evidence [10, 15]. Certainty will be considered either as high (further research is very unlikely to change our confidence in the estimate of effect), moderate (further research is likely to have an important impact on our confidence in the estimate of effect and may change the estimate), low (further research is very likely to have an important impact on our confidence in the estimate of effect and is likely to change the estimate) or very low (very uncertain about the estimate of effect). Two authors will independently apply GRADE to assess the certainty of the evidence.

## Discussion

This review will summarise the evidence on the effectiveness of recruitment interventions evaluated in non-randomised studies. To the best of our knowledge, this is the first systematic review to attempt to collate what is likely to be a substantial body of research. This lack of collation has meant that these studies are essentially lost to trialists when developing their recruitment strategies. We are under no illusions with regard to the likely quality of many or most of these studies for estimating effect; it is likely to be low or very low. However, there are so many of these studies that we are reasonably optimistic that we will be able to pool some studies, apply GRADE and find encouraging results for at least some interventions, which if not sufficiently strong in their own right may nevertheless suggest interventions worth evaluating in future randomised studies. It is also worth remembering that high-quality evidence from randomised evaluations of trial recruitment interventions is currently extremely sparse, which is remarkable given how central recruitment is to trial success [9]. Whilst it may seem like a good idea to combine this review with the Cochrane review of randomised evaluations of recruitment strategies [9], there is a possibility that the outcome of this review is to make clear that non-randomised evaluations are of little use. Should this be the case, the time and effort of doing a combined review now cannot be justified. However, and if appropriate, the two reviews may be combined in the future to produce a full review of recruitment strategies encompassing both randomised and non-randomised evaluation methods.

The results of this review will add to the current evidence base to inform trialists of the most effective methods to recruit participants and will inform researchers of the value (good or bad) of attempting to evaluate interventions in non-randomised ways.

This work is part of the Trial Forge initiative [16] to improve trial efficiency.

## Abbreviations

CINAHL, Cumulative Index to Nursing and Allied Health Literature; CONSORT, Consolidated Standards of Reporting Trials; EMBASE, Excerpta Medica dataBASE; GRADE, Grading of Recommendations Assessment, Development and Evaluation; HTA, Health Technology Assessment; MEDLINE, Medical Literature Analysis and Retrieval System Online; MRC, Medical Research Council; NIHR, National Institute of Health Research; PRISMA, Preferred Reporting Items for Systematic Reviews and Meta-Analyses; RCT, randomised controlled trial; ROBINS-I, Risk Of Bias In Non-randomized Studies - of Interventions; SWAT, Study Within A Trial

## Additional file

Additional file 1: PRISMA-P 2015 checklist. The PRISMA framework will be used to ensure the transparent reporting of this systematic review including the flow diagram. (DOC 99 kb)
